# Enhanced Aggression, Reduced Self-Grooming Behavior and Altered 5-HT Regulation in the Frontal Cortex in Mice Lacking Trace Amine-Associated Receptor 1 (TAAR1)

**DOI:** 10.3390/ijms232214066

**Published:** 2022-11-15

**Authors:** Ilya S. Zhukov, Inessa V. Karpova, Nataliya A. Krotova, Ilya Y. Tissen, Konstantin A. Demin, Petr D. Shabanov, Evgeny A. Budygin, Allan V. Kalueff, Raul R. Gainetdinov

**Affiliations:** 1 Institute of Translational Biomedicine, St. Petersburg State University, University nab. 7/9, 199034 St. Petersburg, Russia; 2 Institute of Experimental Medicine, Acad. Pavlov str. 12, 197376 St. Petersburg, Russia; 3 Almazov National Medical Research Centre, Ministry of Healthcare of Russian Federation, 197341 St. Petersburg, Russia; 4 Neurobiology Program, Sirius University of Science and Technology, 354340 Sochi, Russia; 5 Laboratory of Preclinical Bioscreening, Granov Russian Research Center of Radiology and Surgical Technologies, Ministry of Healthcare of Russian Federation, 197758 St. Petersburg, Russia; 6 Neurobiology Laboratory, Ural Federal University, 620002 Yekaterinburg, Russia; 7 Laboratory of Cell and Molecular Biology and Neurobiology, School of Biological and Medical Physics, Moscow Institute of Physics and Technology, 141701 Moscow, Russia; 8 St. Petersburg University Hospital, St. Petersburg State University, 199034 St. Petersburg, Russia

**Keywords:** TAAR1, gene knockout, aggression, grooming, serotonin, frontal cortex, dopamine, resident-intruder

## Abstract

The Trace Amine-Associated Receptor 1 (TAAR1) is one of the six functional receptors belonging to the family of monoamine-related G protein-coupled receptors (TAAR1-TAAR9) found in humans. However, the exact biological mechanisms of TAAR1 central and peripheral action remain to be fully understood. TAAR1 is widely expressed in the prefrontal cortex and several limbic regions, interplaying with the dopamine system to modulate the reward circuitry. Recent clinical trials suggest the efficacy of TAAR1 agonists as potential novel antipsychotic agents. Here, we characterize behavioral and neurochemical phenotypes of TAAR1 knockout mice, focusing on aggression and self-grooming behavior that both strongly depend on the monoaminergic signaling and cortico-striatal and cortico-limbic circuits. Overall, we report increased aggression in these knockout mice in the resident-intruder test, accompanied by reduced self-grooming behavior in the novelty-induced grooming test, and by higher cortical serotonin (5-HT) tissue levels. Further studies are necessary to explore whether TAAR1-based therapies can become potential novel treatments for a wide range of neuropsychiatric disorders associated with aggression.

## 1. Introduction

Trace amines represent a group of endogenous biogenic amines, such as β-phenylethylamine, p-tyramine, tryptamine, p-octopamine and others [1] that, albeit close to dopamine (DA), serotonin (5-HT) and norepinephrine (NE) structurally, are found in much smaller quantities (often 100 times lower) in the brain [2]. Although trace amines have initially been viewed as ‘false’ neurotransmitters, only indirectly modulating the function of classical monoamines, a family of monoamine-related G protein-coupled receptors (GPCRs) that can be activated by trace amines, termed ‘trace amine-associated receptors’ (TAARs), have later been discovered [3,4,5]. There are currently nine TAAR genes found in mammals, with three of them being pseudogenes in humans [6]. TAAR1 is presently one of the most investigated TAARs, and plays an important role in the central and peripheral nervous systems. For example, TAAR1 is involved in the reward and limbic networks, and is abundantly expressed in the cortex and other key brain regions [6].

While initially suggested as an animal model of schizophrenia [1], TAAR1 knockout (KO) mice demonstrate not only enhanced responses to amphetamine and deficit in sensorimotor gating [7], but also show increased impulsivity and altered wake-sleep cycle [8]. Furthermore, pharmacologic activation of TAAR1 can lead to antipsychotic, antidepressant, procognitive, anti-obsessive, anti-addictive and sleep-modulating effects, suggesting targeting of TAAR1 as a new multimodal therapeutic tool for a wide variety of neuropsychiatric disorders [9,10]. The first developed TAAR1 agonists, RO6889450 (Ralmitaront) and SEP-363856 (Ulotaront), have already been tested in phase II clinical trials for the treatment of schizophrenia, with Ulotaront currently being tested in phase III clinical trials [11].

Notably, patients with several neuropsychiatric conditions, including dementia, schizophrenia, bipolar depression and various neurodevelopmental disorders, demonstrate increased tendency toward violent behaviors [12,13,14,15]. Mounting preclinical evidence links dopamine D1 and D2 receptors in the ventral striatum to the reward and impulsive aspects of aggression, respectively [16,17]. The important role of serotonin neurotransmission in aggressive behaviors is also well established [18]. Moreover, the variation in genes modulating the serotonergic system, as well as inadequate responses to environmental stressors, contribute to negative emotionality and escalate aggressive behaviors [19,20]. Because TAAR1 can modulate serotonin regulation [21] and D2 dopamine receptor function by forming a heteromer receptor complex [1], examining whether TAAR1 genetic ablation in mice may affect their aggressive behavior becomes necessary.

Self-directed grooming is a complex, patterned evolutionally-conserved behavior that is mainly mediated by dopamine transmission within the ventral striatum [22]. Importantly, aberrant self-grooming behavior is commonly observed in animal models of various neuropsychiatric disorders, including attention deficit hyperactivity disorder (ADHD), autism, neurodegenerative and affective pathologies and especially obsessive-compulsive disorder (OCD), all associated with central dopaminergic deficits [23]. Here, we characterize neurochemical and behavioral phenotypes of TAAR1-KO mice, focusing on their aggressive and self-grooming behavior that both rely strongly on monoaminergic signaling in the cortico-striatal and corticolimbic circuits.

## 2. Results

### 2.1. Behavioral Phenotypes

**Figure 1:**
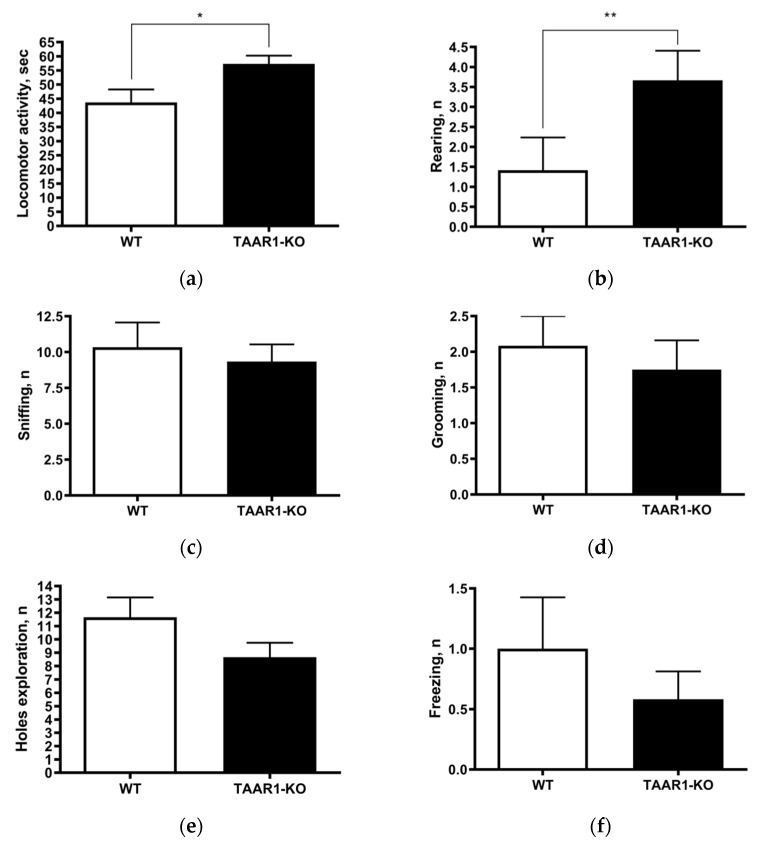
Circular open-field testing data show increased locomotor activity ((**a**); wild type (WT) = 43.67 ± 4.62, vs. TAAR1 knockout (KO) = 57.33 ± 2.93, *p* = 0.0271) and more vertical rearing ((**b**); WT = 1.42 ± 0.82, vs. TAAR1-KO = 3.67 ± 0.74, *p* = 0.0061), but unaltered sniffing, self-grooming, freezing and hole exploration (**c**–**f**) in TAAR1-KO vs. WT control mice. Data are presented as mean ± SEM (n = 12). * *p* < 0.05, ** *p* < 0.01 vs. control, Mann–Whitney U-test.

To evaluate the general behavioral profile of individually housed TAAR1-KO mice, we first tested their behaviors in the circular open-field test. In this test (Figure 1a–f), the TAAR1-KO group showed significantly higher vertical rearing (*p* = 0.0271) and locomotor activity (*p* = 0.0061), but unaltered freezing, self-grooming, sniffing and hole exploration endpoints (Figure 1c–f), compared to the wild type (WT) controls.

**Figure 2:**
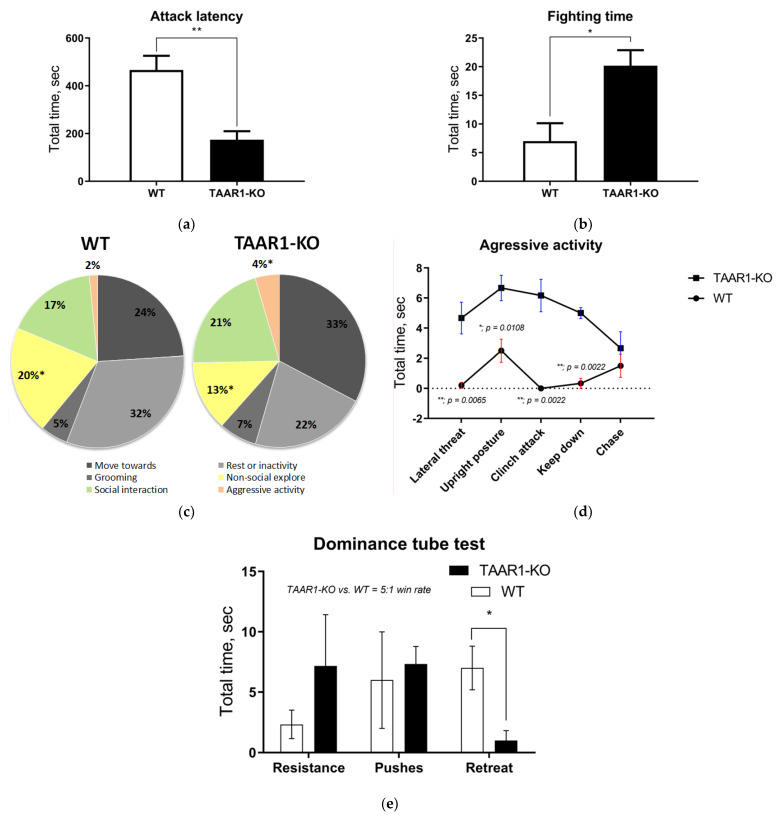
The resident–intruder (**a**–**d**) and the dominance tube (**e**) testing data show that TAAR1 knockout (KO) mice displayed more aggressive and dominant behavior vs. the wild type (WT) control mice. The TAAR1-KO group demonstrated significantly shorter attack latency ((**a**); WT = 466 ± 59.67, vs. TAAR1-KO = 174 ± 35.53, *p* = 0.0087) and longer fighting time ((**b**); WT = 7 ± 3.14, vs. TAAR1-KO = 20 ± 2.71, *p* = 0.03), as well as less non-social exploration ((**c**); WT = 92 ± 5.27, vs. TAAR1-KO = 58 ± 8.81, *p* = 0.015), increased aggressive activity (**d**) and fewer retreats in the dominance tube test ((**e**), WT = 7 ± 1.8, vs. TAAR1-KO = 1 ± 0.82, *p* = 0.035). Data are presented as mean ± SEM (n = 6). * *p* < 0.05, ** *p* < 0.05 vs. control, Mann–Whitney U-test.

In the resident–intruder and dominance tube tests, TAAR1-KO mice demonstrated increased aggression and social dominance (Figure 1 and Figure 2), as assessed by reduced latency to the first attack (*p* = 0. 0087), a longer fighting duration and lower non-social exploration (*p* = 0.0152). In general, the TAAR1-KO resident mice displayed more attacks (Figure 1d), whereas the WT group mainly showed fighting-avoidance behavior. In the dominance tube test, TAAR1-KO mice won five times more often than the WT group, also showing fewer retreats (Figure 2e; *p* = 0.0346, see additional data and videos in the Supplementary Materials, Figure S1 and Video S1).

**Figure 3:**
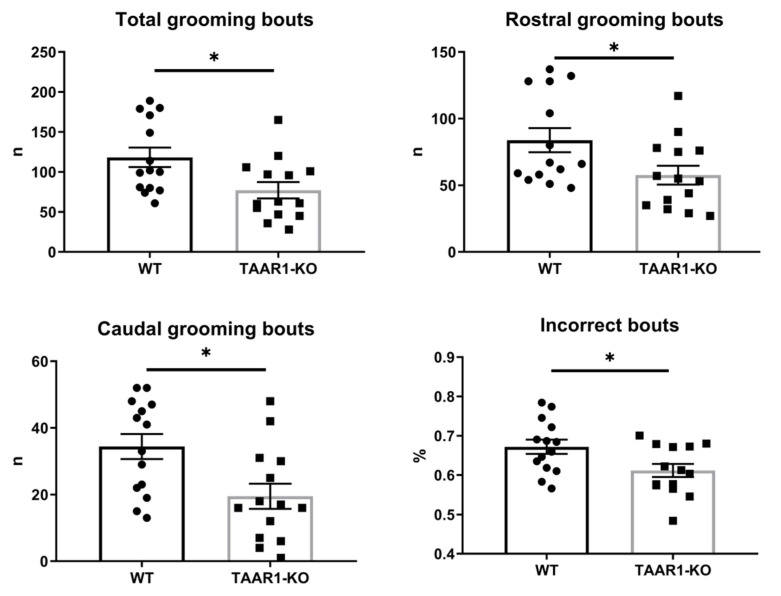
The TAAR1 knockout (KO) mice display altered self-grooming behaviors and aberrant patterning. WT—wild type control mice. Data are presented as mean ± SEM (n = 14). * *p* < 0.05, vs. control Mann–Whitney U-test.

**Figure 4:**
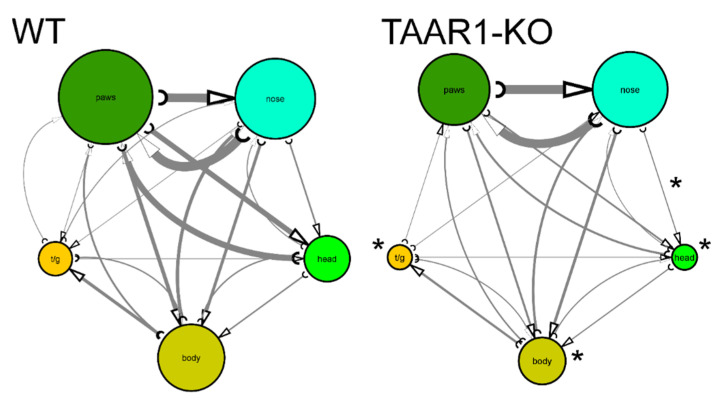
Comparative self-grooming microstructure analyses of TAAR1 knockout (KO) mice vs. wild type (WT) control group in the grooming test (n = 14 per group). The diameter of circles and line thickness reflect mean frequency of grooming bouts or transitions, respectively. All grooming bouts, but only ‘correct’ grooming transitions adhering to the cephalo-caudal progression (paws > face > head > body > tail/genitals) were statistically assessed. * *p* < 0.05 vs. control, Mann–Whitney U-test. WT—wild type control mice.

Analyzing mouse self-grooming behavior and its microstructure, we found more organized and less chaotic microstructure in the TAAR1-KO mice, including fewer incorrect transitions (%), grooming episodes, rostral and caudal bouts, as well as fewer head, body and tail grooming bouts, and fewer nose-to-head transitions among transitions within natural cephalo-caudal progression (Figure 3 and Figure 4, Table 1). No significant differences were observed for other behavioral parameters of mouse self-grooming behavior (Figure 3 and Figure 4, Table 1).

### 2.2. Neurochemical Analyses

**Figure 5:**
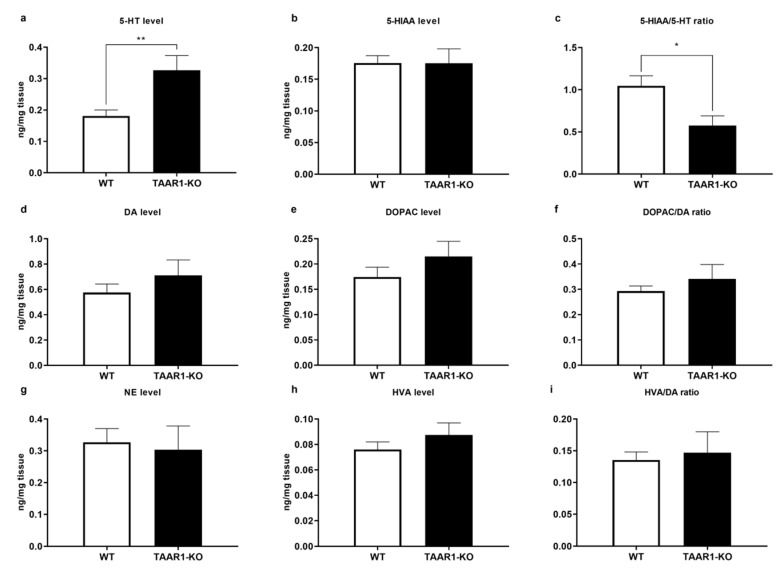
Altered 5-HT neurotransmission in the TAAR1 knockout (KO) mouse cortex. Panels show cortical levels of 5-HT, 5-HIAA and turnover, assessed by the 5-HIAA/5-HT ratio (**a**–**c**), tissue level of dopamine (DA), 3,4-dihydroxyphenylacetic acid (DOPAC) and turnover, assessed by the DOPAC/DA ratio (**d**–**f**), and tissue level of NE, homovanillic acid (HVA) and turnover, assessed by the HVA/DA ratio, in the WT and TAAR1-KO mice (**g**–**i**). Cortical 5-HT level was higher in TAAR1-KO mice (**a**), who also had lower 5-HIAA/5-HT ratio (**c**). Data are presented as mean ± SEM (WT n = 18; TAAR1-KO n = 11). * *p* < 0.05, ** *p* < 0.05 vs. control, Mann–Whitney U-test. Extended data are presented in Supplementary Materials (Table S1).

As shown in Figure 5a–i, the cortical serotonin (5-HT) level was higher in TAAR1-KO mice (Figure 5a; *p* = 0.0021), with unaltered 5-hydroxyindoleacetic acid (5-HIAA) levels (Figure 5b), but impacted 5-HT turnover rate, expressed as the 5-HIAA/5-HT ratio (Figure 5a; *p* = 0.0146). Other neurochemical parameters in different brain structures were unaffected (Supplementary Table S1).

## 3. Discussion

Overall, the present study showed the role of TAAR1 in mouse aggressive behavior and self-grooming syntax, paralleled by dysregulation of 5-HT in the prefrontal cortex, collectively suggesting TAAR1-based therapies as a novel approach to reduce pathological aggression observed in various neuropsychiatric conditions. Indeed, while TAARs are emerging as new multimodal targets in psychopharmacotherapy, this is unsurprising because TAAR1 can modulate DA, 5-HT and glutamate signaling [9]. A key aspect of TAAR1–based therapies involves the regulation of D2 receptors via the heteromer-dependent AKT/GSK3 signaling pathway [1,24,25].

Importantly, aggressive behavior depends on a complex interplay between the dopaminergic and serotonergic cortico-striatal and cortico-limbic signaling [26], and multiple studies implicate DA in aggressive behaviors via D2 receptor inhibition of the gamma aminobutyric acid (GABA)-ergic neurons in various brain regions [27]. Brain serotonin also plays a key role in aggressive behaviors and related psychopathologies. For example, tryptophan hydroxylase 2 (Tph2) KO mice and rats display increased aggressiveness, compared to WT controls [19,28]. Further untangling the complex nature of behavioral regulation of aggression, our findings suggest that trace amines and TAARs can also contribute to this regulation.

The present study found overt changes in the open field test, as the TAAR1-KO mice show higher locomotor and vertical rearing activity. However, it is possible that social isolation stress during testing exacerbated these behavioral manifestations, because such moderate hyperactivity was not consistently observed in non-isolated TAAR1-KO animals [1,6,29]. Furthermore, more remarkable changes were observed in the resident-intruder and the domination tube tests, paralleled by changes in cortical 5-HT levels.

On the one hand, excessive agonist concentrations at 5-HT1A and 5-HT1B receptors in the medial prefrontal cortex or lateral septum have already been linked to mouse aggressive behavior [30], and altered 5-HT and NE in the prefrontal cortex elicit pathological aggressive phenotypes in mice [31]. Moreover, stimulation of the prefrontal cortex reduces aggressive intentions in patients clinically [32]. In line with this, we observed that TAAR1 genetic knockout also affects 5-HT concentration in the prefrontal cortex, likely due to increased firing of serotonergic neurons [33]. Intriguingly, as with D2 DA receptors [34], the heteromerization of TAAR1 with 5-HT1B receptors [35] may also impact autoreceptor-mediated presynaptic regulation of 5-HT function. On the other hand, while testosterone signaling regulates sexual and aggressive social behavior in mice [36], TAAR1-KO mice display minimal alterations in sexual motivation and testosterone levels [37]. Collectively, this suggests that TAAR1-based therapies may exert precise effects on aggression through neuromodulatory mechanisms, without directly affecting endocrine regulation and sexual motivation.

Self-grooming represents another critical behavioral phenotype for studying a wide range of animal psychiatric models [23]. An overall intensity of self-grooming activity and its specific patterning often are the most valuable and most sensitive grooming-related phenotypes [23]. Briefly, the former focuses on studying self-grooming duration or the number of self-grooming bouts in the test, whereas the latter examines the evolutionarily conserved cephalocaudal progression of self-grooming (i.e., paws > face > head > body > tail/genitals). Different combinations of those two phenotypes contribute to various pathological phenotypes, including likely neurological deficits (when both indices are reduced), OCD-like states (when both are increased) and affective-like states (when the two indices may display varying changes) [23].

Overall, the results of the grooming test here (Figure 3 and Figure 4) support global reduction in self-grooming behavior in TAAR1-KO mice. While reduced caudal self-grooming may support an anxiety-like profile [23] already reported in TAAR1-KO mice [38,39], the parallel inhibition of rostral grooming suggests a rather global reduction of this behavior, perhaps paralleling concomitant increase in the locomotor activity. Alternatively, reduced self-grooming activity is often observed in rodent models of depression and/or neurodegenerative disorders [40], potentially implicating a complex combination of affective and other pathological states in TAAR1 dysregulation.

Interestingly, Wistar-Kyoto rats show low self-grooming activity that is recovered after exposure to serotonergic antidepressant clomipramine [41], supporting a complex interaction between the serotonergic system and aberrant self-grooming activity observed here. Finally, lower self-grooming behavior is also observed following ventral pallidum (VP) lesions [42], thus potentially also implicating both glutamatergic and cholinergic dysfunction as well, albeit out of the scope of the present study.

Uncontrollable violence and aggressive behavior are a critical social and medical problem. The present study demonstrated that the TAAR1 receptor may play an important role in the orchestration of aggressive reactions through altering cortical 5-HT levels and turnover. Further studies in this field are needed to better understand molecular pathways of the TAAR1-DA-5-HT regulation. Nevertheless, our research for the first time revealed neurobiological association of trace amines and their receptors with aggressive behavior. Although DA and 5-HT have long been established as major modulators of aggression, trace amines may represent another key neurobiological mechanism of aggression, clinically relevant to a wide range of psychiatric disorders. Thus, future detailed studies with TAAR1-selective pharmacological tools are necessary to further support the role of TAAR1 in aggression. Our findings also suggest potential pharmacological perspectives of TAAR1-based drugs in the context of aggressive behavior treatment. Taken together, enhanced aggression, reduced self-grooming behavior and altered 5-HT in the frontal cortex of TAAR-KO mice, such as reported here, further implicate trace amines and their receptors in central regulation of complex CNS functions and behaviors.

## 4. Materials and Methods

### 4.1. Animals

All animal studies were performed according to the guidelines of the Ministry of Health of the Russian Federation and the principles adopted by the Federation of European Laboratory Animal Science Associations (FELASA) and the Russian Laboratory Animal Science Association (RusLASA) for the welfare of laboratory animal use. All experiments reported here were approved by the Saint Petersburg State University Ethical Committee for Animal Research (approval 131-03-1 of 07.16.2020). The WT (+/+) and TAAR1-KO (−/−) mice were derived by crossing (for >20 generations) heterozygous TAAR1 +/− C57BL6/129SvJ mice. Experimental adult male mice (35 ± 5 weeks old) were housed 3–5 per cage (170 × 143 × 363.5 mm) before isolation for 4 months, and maintained under standard laboratory conditions (with environmental enrichment and room temperature and humidity of 21 ± 5 °C and 40–70%, respectively) with food (Mucedola S.R.L., Settimo Milanesse, Italy) and water ad libitum. The grooming test was performed between 18:00–21:00 h during the light phase. The resident-intruder and the tube dominance tests were performed during the dark phase between 21:00–01:00 h. The mice were acclimated to the experimental room for at least 1 h prior to behavioral testing.

### 4.2. Behavioral Assays

The open field test, used here to measure mouse locomotor and exploratory activity (n = 12), was a gray plastic circular arena (67 cm in diameter) with 13 holes (1 cm in diameter) in the arena floor. The mice were individually placed at the center of the arena, and their spontaneous exploration activity was video-recorded for 10 min, scoring the locomotor activity (distance traveled, s), the number of freezing and sniffing, total duration of self-grooming (s), the number of vertical rears and holes inspected. Between the animals, the arena was cleaned with 3% hydrogen, to eliminate olfactory cues. All endpoints of recorded behavior were scored manually frame-by-frame by a highly experienced scorer blinded to the genotype.

The resident-intruder paradigm was used here to evaluate territorial aggression in mice [43]. Isolated (for 4 months) adult TAAR1-KO and WT male mice (residents, n = 6) of equal weight were placed for 10 min with socially housed intruder mice of the CD-1 strain. Every second of recorded behavior was scored manually frame-by-frame blinded to the genotype, scoring the duration (s) of social interaction (anogenital, tail, body and nose sniffing), non-social exploration (vertical rearing, wall-supported vertical rears, burying, sniffing and cage sniffing) and aggressive activity (lateral threats, upright postures, clinch attacks, keeping down, chasing), as well as some additional behavioral endpoints, such as approach, rest/inactivity and self-grooming (see detailed protocol in the Supplementary Materials).

Three days after the resident-intruder test, the same groups were tested in pairs (WT vs. TAAR1-KO) in the tube dominance test (n = 6) [44], scoring the duration (s) of resistance, pushes, still and retreat (see video samples and additional Figures in the Supplementary Materials).

The grooming test (GT) was used here to characterize both basic mouse self-grooming behavior and its complex microstructural behavioral patterns. Briefly, mice (n = 14) were individually placed into a transparent glass cylindrical jar (20 cm in diameter, 45 cm height) and their self-grooming was recorded for 10 min using an Apple iPhone SE (1st generation) video-camera (Apple Inc., Cupertino, CA, USA). Recorded self-grooming behavior was then scored manually frame-by-frame by a highly experienced scorer blinded to genotype, assessing the number of total grooming bouts, rostral grooming bouts, caudal grooming bouts, as well as paw, face, head, body and tail grooming episodes. To further analyze self-grooming microstructure, the number of grooming transitions between different body parts (e.g., nose to head, head to tail) were also assessed here. Any transition between grooming stages that violated normal cephalo-caudal progression (paws > face > head > body > tail) was considered incorrect, yielding the percentage of incorrect transitions in total transitions. We also compared globally the number of transitions for each cephalo-caudal progression and generated ethograms, to better represent grooming microstructural sequential patterns according to [23].

### 4.3. HPLC Measurements of the Monoamines Tissue Content

Monoamines examined here included NE, DA, 5-HT, 3,4-dihydroxyphenylacetic acid (DOPAC), homovanillic acid (HVA) and 5-hydroxyindoleacetic acid (5-HIAA), determined by reverse-phase high-performance liquid chromatography on a Shimadzu LC-20 Prominence chromatograph (Shimadzu, Japan) with a Decade Elite electrochemical detector (Antec, The Netherlands). The chromatographic system included a Rheodyne 7125 injector (Rheodyne LLC, USA) with a 20 µL loop for the sample application and a Phenomenex column (4.6 × 150.0 mm) with a Sphere Clone 5 u ODS (2) sorbent (Phenomenex Inc., USA). Several brain regions (olfactory tubercle, hippocampus, striatum and cerebral cortex) were isolated from the left hemisphere and dissected on ice, frozen in liquid nitrogen and stored at −80. Tissue samples were homogenized in liquid nitrogen at −198° C with CryoMill (Retsch, Germany).

The samples were then suspended in 0.1 M hydrochloric acid (HCl) as follows: samples of the striatum in 50 µL, hippocampus or olfactory tubercle in 100 µL, cortex of the cerebral hemispheres in 150 µL. They were then centrifuged for 20 min with an acceleration of 14,000× *g* at +6 °C. The supernatant was collected into clean plastic tubes and stored until analysis at a temperature of −80 °C. The samples were re-thawed on the day of analysis, after which they were centrifuged again (14,000× *g*, 20 min, at +6 °C) to avoid the possible ingress of the remaining sediment particles into the chromatographic system [45], and analyzed at +30 °C, potential of +0.70 V, mobile phase containing 5.5 mM citrate-phosphate buffer with 0.7 mM octane sulfonic acid, 0.5 mM and 6.5% acetonitrile (pH 3.0), with the elution rate 0.8 mL/min and a 20 min analysis time (please see full table in the Supplementary Materials, Table S1).

### 4.4. Statistical Analyses

Statistical analysis was performed with GraphPadPrism 6.0 (GraphPad Software, USA) using the non-parametric Mann–Whitney U-test to compare the WT and TAAR1-KO groups (*p* set at <0.005).

## Figures and Tables

**Table 1:** Summary of statistical results of TAAR1 knockout (KO)-induced behavioral changes in mouse self-grooming test and self-grooming microstructure analysis. WT—wild type control mice. Data are presented as mean ± SEM (n = 14). * *p* < 0.05 vs. control, Mann–Whitney U-test.

Endpoint	WT	TAAR1-KO	U	*p* Value
Incorrect transitions, %	0.67 ± 0.02	0.6118 ± 0.01679 *	50	0.0274
Total grooming bouts, n	118.3 ± 12.2	77.14 ± 10.22 *	45.4	0.0145
Rostral grooming bouts, n	83.9 ± 9.1	57.64 ± 7.077 *	51	0.0303
Caudal grooming bouts, n	34.4 ± 3.8	19.5 ± 3.781 *	44.5	0.0127
Interrupted grooming bouts, %	0.40 ± 0.04	0.3854 ± 0.06415	70	0.2057
Paws grooming bouts, n	37.57 ± 4.715	25.36 ± 2.946	57	0.0603
Nose grooming bouts, n	31.14 ± 2.721	27.21 ± 3.551	75	0.3004
Head grooming bouts, n	15.14 ± 3.572	5.071 ± 1.777 *	43	0.0099
Body grooming bouts, n	24.86 ± 2.957	14.17 ± 3.153 *	48.5	0.0217
Tail grooming bouts, n	9.571 + 1.797	4.786 + 1.1 *	52	0.0333
Paws to nose transitions, n	16.5 ± 1.504	16.64 ± 2.269	95.5	0.9185
Nose to head transitions, n	2.214 ± 0.5565	1 ± 0.4804 *	56.5	0.0478
Head to body transitions, n	1.929 ± 0.5494	1.214 ± 0.6305	58.5	0.0595
Body to tail transitions, n	5.357 ± 1.137	3.357 ± 0.7956	70.5	0.211

## Data Availability

All of the data are presented in the article and Supplementary Materials. No additional data are reported.

## Supplementary Materials

The supporting information can be downloaded at: https://www.mdpi.com/article/10.3390/ijms232214066/s1.
