# Supplementary material for: Enhanced Aggression, Reduced Self-Grooming Behavior and Altered 5-HT Regulation in the Frontal Cortex in Mice Lacking Trace Amine-Associated Receptor 1 (TAAR1)
Source: Int J Mol Sci. 2022 Nov 15;23(22):14066. doi: 10.3390/ijms232214066 (PMC9695497; doi:10.3390/ijms232214066)
Supplement: Supplementary file 1 [file ijms-23-14066-s001.zip › Supplementary Materials.pdf]

The resident-intruder test protocol.

The resident-intruder paradigm was used here to evaluate the territorial aggression of TAAR1-KO and WT mice. It was based on the original rat protocol (Koolhaas et.al 2013) with the addition of extended endpoints. Only resident-mice endpoints were counted without defensive behavior. Comprehensive endpoints of mice resident-intruder tests are needed to cover the entire repertoire of behavioral activity.

1) TAAR1-KO and WT males were isolated (4 months). Female companions in the resident cage were not used in the current experiment.
2) Bedding of the resident cage was not cleaned for 1 week before the experiment.
3) Resident-intruder tests were conducted during the dark phase from 21:00 to 01:00. TAAR1-KO and WT mice of equal weight were placed with socialized intruder mice (CD-1).
4) The video was recorded for 10 min using Apple iPhone SE (1st generation) video camera (Apple Inc., Cupertino, USA).
5) After the experiment every second of recorded behavior was scored manually frame-by-frame blinded to the genotype, scoring the duration (in seconds) of social interaction (anogenital sniffing, tail sniffing, body sniffing, nose sniffing), non-social exploration (rearing, wall stand, burying, sniffing, cage sniffing), and aggressive activity (lateral threat, upright posture, clinch attack, keep down, chase), as well as some additional behavioral endpoints, such as approach, rest/inactivity, and grooming.

Supplementary Figure S1. Additional visualization of resident-intruder test.

A.

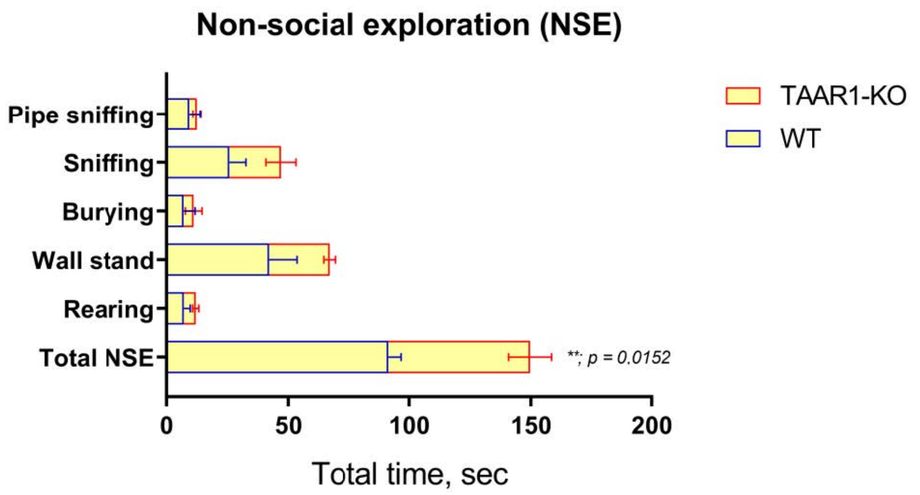

B.

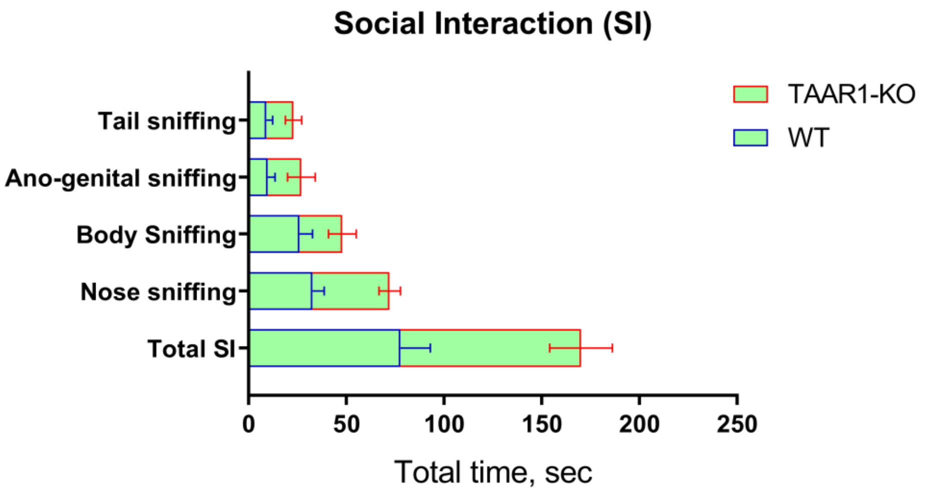

C.

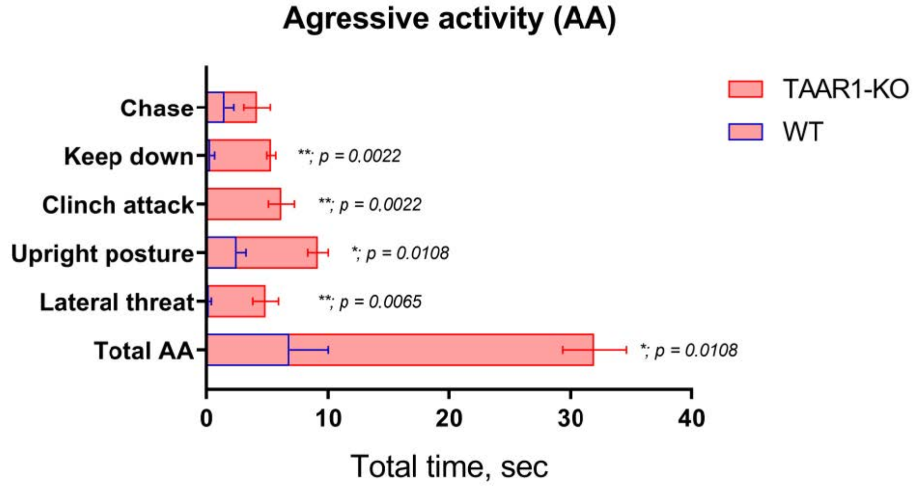

D.

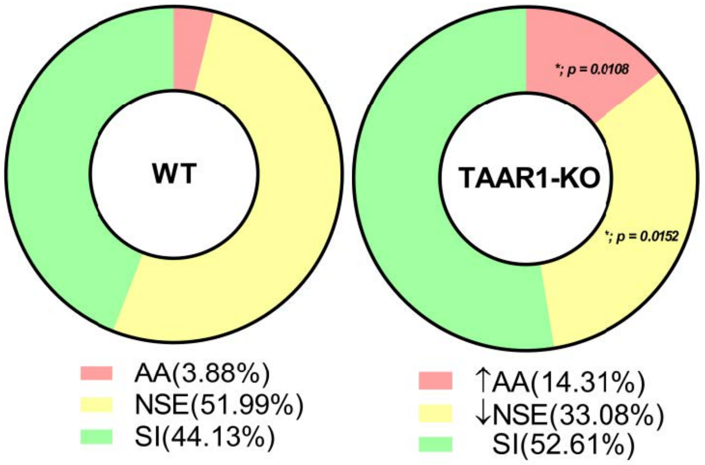

E

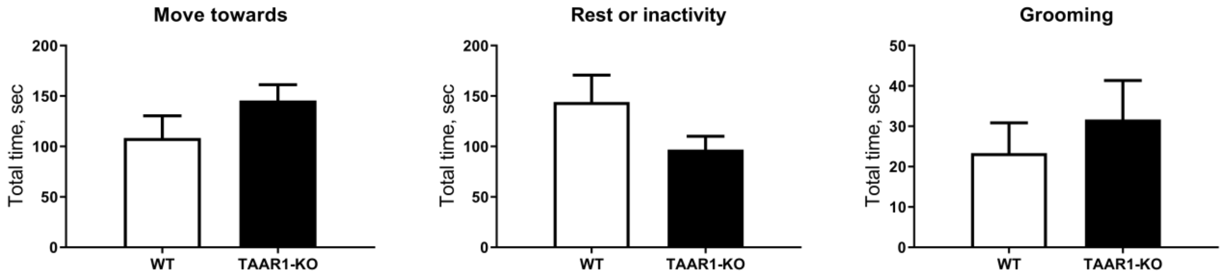

Total non-social interaction (NSE) decreased in TAAR1-KO mice (Figure SF 1 A, *; p = 0.0152).There are no significant differences in social interaction (SI) (Figure SF 1 B). Aggressive activity (AA) is significantly increased in TAAR1-KO mice (Figure SF 1 C). Additional visualization of TAAR1-KO resident-intruder test profile (Figure SF 1 D). Other parameters such as move towards, rest or inactivity and grooming had minimal alterations (Figure SF 1 E). Data are mean ± SEM. *p<0.05, **p<0.05 vs. control Mann-Whitney U-test.

Supplementary Table S1. The full table of HPLC measurements of the monoamines tissue content.

Cortex (ng/mg tissue)							
Genotype	WT			TAAR1-KO			P value
Parameters	Mean	±SEM	N	Mean	±SEM	N	
5-HT	0.181	0.01902	18	0.04696	0.124	10	0.0021**
5-HIAA	0.1757	0.01162	18	0.1753	0.02274	10	0.9812
5-HIAA/5-HT	1.045	0.1207	17	0.5753	0.1152	11	0.0146*
DA	0.5756	0.06709	18	0.7108	0.1219	7	0.3261
DOPAC	0.1746	0.01929	18	0.215	0.02993	11	0.2564
DOPAC/DA	0.2933	0.01993	17	0.3416	0.05648	7	0.4181
NA	0.3269	0.04331	18	0.3034	0.07465	6	0.6261
HVA	0.07602	0.006029	18	0.08747	0.009529	11	0.1733
HVA/DA	0.1354	0.01279	16	0.147	0.03283	10	0.9221

Striatum (ng/mg tissue)							
Genotype	WT			TAAR1-KO			P value
Parameters	Mean	±SEM	N	Mean	±SEM	N	
5-HT	0.9151	0.1008	15	0.8347	0.124	11	0.6461
5-HIAA	0.3308	0.02837	15	0.2866	0.02995	11	0.3051
5-HIAA/5-HT	0.4025	0.05374	14	0.4274	0.08103	11	0.9786
DA	5.03	0.854	12	5.888	0.9194	8	0.4727
DOPAC	0.728	0.06606	14	0.5932	0.07528	10	0.2408
DOPAC/DA	0.1251	0.0277	13	0.1249	0.02238	10	0.7844
NA	0.1058	0.01661	16	0.09698	0.01916	11	0.7897
HVA	0.37	0.02341	14	0.3539	0.05418	11	0.9786
HVA/DA	0.05344	0.01326	17	0.06549	0.01578	11	0.5471

Hippocampus (ng/mg tissue)							
Genotype	WT			TAAR1-KO			P value
Parameters	Mean	±SEM	N	Mean	±SEM	N	
5-HT	0.7002	0.06137	18	0.6969	0.1105	11	0.6423
5-HIAA	0.3476	0.02734	18	0.3556	0.03271	11	0.9824
5-HIAA/5-HT	0.5199	0.04904	18	0.5723	0.07152	11	0.5501
DA	0.2669	0.04233	18	0.2916	0.04018	11	0.5801
DOPAC	0.1604	0.01754	18	0.1971	0.0302	11	0.4923
DOPAC/DA	1.004	0.1963	18	0.823	0.1504	11	0.8424
NA	0.6041	0.0763	18	0.6411	0.06337	11	0.5279
HVA	0.1027	0.01233	18	0.1105	0.01226	11	0.4647
HVA/DA	0.4658	0.0932	16	0.3713	0.04953	10	0.8564

Olfactory tubercle (ng/mg tissue)							
Genotype	WT			TAAR1-KO			P value
Parameters	Mean	±SEM	N	Mean	±SEM	N	
5-HT	0.6541	0.04782	17	0.7368	0.07392	11	0.2441
5-HIAA	0.3839	0.03302	18	0.3689	0.03137	11	0.5726
5-HIAA/5-HT	0.6295	0.03674	18	0.5419	0.05237	11	0.1223
DA	3.182	0.4222	16	2.39	0.5065	11	0.0453
DOPAC	0.2233	0.01568	17	0.2188	0.01369	11	0.8622
DOPAC/DA	0.07367	0.009257	17	0.1017	0.01062	10	0.0638
NA	0.6639	0.05255	18	0.7053	0.07561	10	0.9063
HVA	0.2342	0.01701	17	0.2649	0.03011	11	0.329
HVA/DA	0.07367	0.009257	17	0.1017	0.01062	10	0.0638

Cortical 5-HT level higher in TAAR1-KO mice, 5-HIAA/5-HT ratio decreased in TAAR1-KO. Other parameters have minimal alterations. Data are mean ± SEM. *p<0.05, **p<0.05 vs. control Mann-Whitney U-test.
